# Improving classification accuracy of fine-tuned CNN models: Impact of hyperparameter optimization

**DOI:** 10.1016/j.heliyon.2024.e26586

**Published:** 2024-02-23

**Authors:** Mikolaj Wojciuk, Zaneta Swiderska-Chadaj, Krzysztof Siwek, Arkadiusz Gertych

**Affiliations:** a Faculty of Electrical Engineering, Warsaw University of Technology, Warsaw, Poland; b Department of Surgery, Department of Pathology and Laboratory Medicine, Cedars-Sinai Medical Center, Los Angeles, CA, USA; c Faculty of Biomedical Engineering, Silesian University of Technology, Zabrze, Poland

**Keywords:** Optimization, Hyperparameters, Fine-tuning, Deep learning

## Abstract

The immense popularity of convolutional neural network (CNN) models has sparked a growing interest in optimizing their hyperparameters. Discovering the ideal values for hyperparameters to achieve optimal CNN training is a complex and time-consuming task, often requiring repetitive numerical experiments. As a result, significant attention is currently being devoted to developing methods aimed at tailoring hyperparameters for specific CNN models and classification tasks. While existing optimization methods often yield favorable image classification results, they do not provide guidance on which hyperparameters are worth optimizing, the appropriate value ranges for those hyperparameters, or whether it is reasonable to use a subset of training data for the optimization process. This work is focused on the optimization of hyperparameters during transfer learning, with the goal of investigating how different optimization methods and hyperparameter selections impact the performance of fine-tuned models. In our experiments, we assessed the importance of various hyperparameters and identified the ranges within which optimal CNN training can be achieved. Additionally, we compared four hyperparameter optimization methods—grid search, random search, Bayesian optimization, and the Asynchronous Successive Halving Algorithm (ASHA). We also explored the feasibility of fine-tuning hyperparameters using a subset of the training data. By optimizing the hyperparameters, we observed an improvement in CNN classification accuracy of up to 6%. Furthermore, we found that achieving a balance in class distribution within the subset of data used for parameter optimization is crucial in establishing the optimal set of hyperparameters for CNN training. The results we obtained demonstrate that hyperparameter optimization is highly dependent on the specific task and dataset at hand.

## 1 Introduction

Developing a machine learning algorithm is a complex task, that involves various components such as selecting the model type, collecting and preparing the dataset, and optimizing hyperparameters of the model. Each of these components plays a crucial role in determining the final performance of the algorithm. Hyperparameter optimization, in particular, is vital for controlling the behavior of a machine learning model by identifying the optimal values for its hyperparameters during the learning process.

The growing interest in hyperparameter optimization is closely tied to the pursuit of creating reliable deep-learning models for numerous applications [1], [2], [3], [4], [5], [6], [7]. Recent studies have demonstrated that adjusting hyperparameters can significantly enhance CNN's model performance [8], [9], [10], [11], [12]. The application of hyperparameter optimization techniques has an impact on (I) the improvement of the CNN performance (by a few percentages), (II) the reduction of method development time (when combined with distributed training for quick parallel searching), and (III) the faster provision of solutions, which is a crucial aspect for businesses.

The hyperparameters generally relate to the network architecture and training methods. The goal of hyperparameter optimization is to determine a set of hyperparameters that, when utilized during CNN training, yield the highest possible classification accuracy on a test set. However, the process of selecting hyperparameters that result in satisfactory CNN performance is not trivial. It often involves evaluating the CNN model multiple times, making it computationally expensive.

In practical terms, exhaustively testing all possible combinations of hyperparameters (e.g., through grid search) can eventually yield an optimal set. Nevertheless, this approach is time-consuming. Fortunately, emerging optimization methods [13], [14], [15], [16] aim to not only produce good training results but also reduce the computational resources required for this purpose.

Setting hyperparameters is not a simple task, and searching through the entire hyperparameter space can be time-consuming, computationally expensive, and inefficient [17]. Various techniques have been developed for hyperparameter optimization, including Random Search, Grid Search, Hessian-free optimization [18], Genetic Algorithms [19], [13], [14], [15], [16], Bayesian Optimization [8], Bayesian Optimization (BOHB) and Hyperband method [20], and the Asynchronous Successive Halving Algorithm (ASHA) [21], [22]. Among these, Random Search and Grid Search are the most popular methods and allow for the implementation of prior knowledge about hyperparameter values.

Grid Search, also known as parameter sweep, is a basic method for hyperparameter optimization that involves exhaustive searching. This approach selects hyperparameters from a multidimensional space using a grid, where the number of hyperparameters determines the dimensionality. For example, with two parameters and three possible values each, there would be a total of nine parameter sets. The coordinates of grid points represent the hyperparameter values, and the spacing between the points (as well as the total number of configurations) is typically defined by the user [19]. The set of possible hyperparameter values can be predefined or selected from a continuous range specified by the user. The number of CNN models trained using the grid method is determined by the number of hyperparameter combinations in the grid. A fundamental disadvantage of this approach is that the number of hyperparameter sets grows rapidly as the number of grid points increases.

Random Search, on the other hand, involves randomly selecting hyperparameter values from the defined hyperparameter space [19]. For discrete hyperparameters, such as the optimization technique used (e.g., gradient descent), a value is chosen from the available options. For continuous hyperparameters, such as learning rate or dropout, a random value is selected from the specified range. Unlike Grid Search, Random Search does not investigate all possible combinations. The number of evaluated CNN models depends on the number of random searches, but it is independent of the size of the hyperparameter space. For example, with two parameters having three possible values each and two random searches, there would be a total of nine possible combinations, but only two parameter sets would be investigated. In this method, domain knowledge or prior information can be used to guide the selection of parameter sets or the hyperparameter space.

ASHA (Asynchronous Successive Halving) is a parametric method that leverages randomly selected sets of hyperparameters, which are then evaluated to identify those that are likely to result in an optimally trained CNN [22], [21]. Unlike Random Search, ASHA updates its knowledge about hyperparameter sets as the CNN training progresses. In this method, a certain number of randomly selected hyperparameter sets are used to train CNN models, with each model trained for a specific number of epochs. Based on the performance achieved by each model, a subset of models is chosen for further training with a longer duration, while the remaining models are saved. This process of selecting the best CNN model is repeated iteratively. When multiple fully trained models exist, the ASHA method generates new hyperparameter sets, evaluates them while considering the previously used sets, and determines which models to include in subsequent iterations. If a new set yields worse CNN results than the previous sets, computational resources may be allocated to continue evaluating the previously rejected sets.

To utilize ASHA, five parameters must be defined: r—minimum number of training epochs, R—maximum number of training epochs, *η*—elimination coefficient, s—early stopping criterion, and $N_{m}$—the number of fully trained models. Importantly, the number of models trained for r epochs is not equal to the number of fully trained models (trained for R epochs) because ASHA continuously updates its knowledge of hyperparameter sets and generates only as many new sets as necessary to continue the process. The main advantages of ASHA are the efficiency of the hyperparameter optimization process and the utilization of limited computational resources.

Bayesian Optimization is an approach that involves multiple steps to optimize hyperparameters. In the initial step, the algorithm randomly generates a certain number of hyperparameter configurations or loads pre-existing ones for evaluation. It also keeps track of past evaluations of CNN models to build a probabilistic classifier that maps hyperparameters to a probability score in the objective function. New hyperparameter configurations are generated based on the results of previous evaluations and previously used configurations. In this method, hyperparameter optimization is achieved by optimizing a surrogate function that is easier to optimize than the actual objective function [23], [12], [24], [25], [26], [27]. By incorporating previous sets of hyperparameters that have yielded promising models in the search, Bayesian Optimization can discover more optimal hyperparameter sets compared to Random Search, and it typically requires fewer iterations. The algorithm needs to strike a balance between exploration and exploitation to find the most optimal set of hyperparameters. It may exploit known areas of the hyperparameter space where satisfactory results have been obtained, while also exploring unknown areas in search of potential improvements. Once a new set of hyperparameters is selected and defined, the CNN model training process is carried out, and the newly found hyperparameters replace those from the previous iteration. The algorithm iteratively updates the hyperparameters until a predefined number of trained models is reached. In this paper, we utilized a variant of Bayesian Optimization with an acquisition function [28]. The choice of new hyperparameter sets is based on the highest probability of achieving better results. In our study, we initially generated four randomly selected sets of hyperparameters using Bayesian Optimization and then proceeded to generate subsequent sets based on acquired knowledge. It is possible to specify a larger number of sets depending on the implementation of Bayesian Optimization and the number of hyperparameters being optimized.

The use of the functional analysis of variance (fANOVA) method in the deep learning domain has been gaining popularity in recent years. It is primarily applied in two main areas: (I) increasing the interpretability of deep learning algorithms [29], and (II) evaluating the importance of hyperparameters in machine learning tasks [30], [31]. Several studies have demonstrated promising results in utilizing fANOVA for hyperparameter optimization in machine learning [30], [32], [33], [34]. As a result, the fANOVA technique has been integrated into complex solutions described in the refs. [35], [36] and employed for hyperparameter optimization in Graph Neural Networks [37] as well as for tuning reinforcement learning parameters [34].

The main contribution of this paper lies in investigating the impact of hyperparameter optimization on the transfer learning of CNN. While existing literature extensively covers the influence of hyperparameter optimization on the improvement of CNNs trained from scratch, the aspect of fine-tuned CNNs has been overlooked, leaving room for further research, which we address in this paper. The key advantage of the fine-tuning approach is the ability to utilize small training datasets and reduce training time when compared to de-novo-trained CNNs. Pretrained CNNs can identify basic features such as borders and shapes in the initial layers, while deeper layers can recognize more abstract features. As a result, pretrained CNNs prove to be efficient in detecting basic features and fine-tuning the last layers is often sufficient to achieve good classification performance on unseen images. Pretrained models also become handy when de-novo training is not possible or would likely result in low CNN accuracy due to limited training instances.

Among the paper's strengths, we can highlight: (I) exploring hyperparameter optimization in transfer learning scenarios, (II) investigating four hyperparameter optimization techniques, and (III) examining the use of subsets of data (both balanced and imbalanced) for the optimization process.

## 2 Materials

The impact of ASHA, Grid Search, Random Search, and Bayesian hyperparameter optimization techniques on the classification performance of CNNs was investigated using three publicly available datasets. These datasets vary in terms of the number of images, class labels, and image sizes. The selected datasets are as follows:

• CIFAR-100 [38]: This well-known dataset consists of 60,000 natural images from 100 various classes, including animals, objects, and plants. All images in this dataset have a fixed size of 32 × 32 pixels.
• Stanford Dogs [39]: This dataset contains 20,580 dog images divided into 120 classes. The images vary in size and often depict dogs facing the camera (dog faces), but there are also images showing humans and dogs or whole dogs. The Stanford Dogs dataset is imbalanced in terms of the number of images per class, image size, and image type within each class.
• MIO-TCD [40]: This dataset focuses on street traffic images and consists of 519,164 labeled images captured by surveillance cameras at different angles. The images are grouped into 11 classes: cars, trucks, and pedestrians. For this study, a class-balanced subset (19,250 images) and a class-imbalanced subset (19,250 images) were distinguished from the labeled set. These subsets were further divided into training, validation, and test sets (Table 1). It is important to note that there is an overlap between the images in the balanced and imbalanced training sets, but the test set is disjointed from these two sets. In both cases, the test set is balanced and includes 500 images from each class, jointly 5500 images.

**Table 1:** Datasets used in the study, where * indicates the subset of the labeled images from the MIO-TCD dataset was used for the method development, **indicates that all test sets were balanced.

Dataset	Number of images	Number of classes	Balanced training set	Number of images per class	Data split		
					Training	Validation	Test**
CIFAR-100	60,000	100	Yes	600	40,000	10,000	10,000
Stanford Dogs	20,580	120	No	148–252	15,504	3,876	1,200
MIO-TCD*	519,164 (all labeled images)	11	No	1,751–260,518	410,931	102,733	5,500
	19,250 (class-imbalanced)	11	No	33–6,936	11,000	2,750	5,500
	19,250 (class-balanced)	11	Yes	1,750	11,000	2,750	5,500

The datasets were split into training, validation, and test cohorts before conducting the experiments. The same subsets resulting from the split were used in each experiment to ensure consistency. Table 1 summarizes information about datasets and subsets used in the study.

## 3 Methods

In this study, the impact of Random Search, Grid Search, Bayesian Optimization, and ASHA hyperparameter optimization methods was investigated on the classification performance of the Xception [41] CNN model (Fig. A.4, supplementary materials) pretrained on ImageNet [42]. The experiments were conducted using Python, specifically utilizing the Keras and TensorFlow libraries, which are commonly used for deep learning tasks.

Hyperparameters can be categorized into model parameters (e.g., pooling size, number of hidden layers), and training parameters (e.g., regularization, learning rate, batch size). For this research, the focus was on six common training hyperparameters divided into three groups: (I) numerical discreet hyperparameters: (a) input image size, (b) batch size, (c) number of fine-tuned CNN layers, (II) numerical continuous hyperparameters: (d) dropout rate, (e) learning rate, and (III) categorical hyperparameters: (f) gradient descent (GD) optimization technique (e.g., Adam, Adadelta, RM-Sprop, SGD). The initial range and type of the hyperparameters are shown in Table 2A.

**Table 2:** Hyperparameters space.

Hyperparameter	A-Initial Range	B-Applied Range	
	All methods	Random Search, ASHA, Bayesian	Grid Search

Learning rate	0.00001–0.1	0.0001–0.03	0.001 & 0.01
Dropout value	0.01–0.4	0.01–0.4	0.2
GD optimization technique	Adam, Adadelta, RMSprop, SGD	Adam, Adadelta, RMSprop, SGD	Adam, RMSprop
Nb of fine-tuned CNN layers	0, 9, 14, 18, 132	9, 14, 18, 132	
Input image size	71, 128, 256	256	
Batch size	16, 32, 64	16	

Our study was motivated by the involvement of expert knowledge and computational resources, which are often needed (but not always available), to manually identify hyperparameters that can lead to an optimally trained CNN model. In practice, the selection of hyperparameters is further complicated by the interplay between parameters on each other, making it difficult to assess their impact a priori, and this may vary depending on the classification task.

To ensure a fair comparison and manage computational resources effectively, the total number of epochs dedicated to hyperparameter optimization for each method was limited to 400. This limit was determined by multiplying the number of hyperparameter sets (16 sets) and the number of epochs the CNN was trained with using a single set of hyperparameters (25 epochs). Thus, the computational budget for the entire experiment amounted to 4 × 400 = 1,600 epochs.

**Figure 1:**
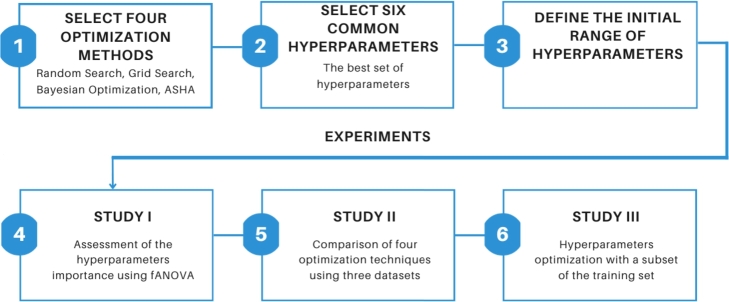
The main steps of the experiment.

Our experiments were conducted in three steps, as shown in Fig. 1. Firstly, we assessed the importance of hyperparameters. Next, we compared four optimization techniques using the CNN trained with CIFAR-1000, Stanford Dogs, and MIO-TCD datasets. Lastly, we investigated the feasibility of utilizing a subset of training data to effectively optimize hyperparameters.

### 3.1 Assessment of hyperparameters importance

To examine the impact of hyperparameter changes on CNN's performance, we employed the functional analysis of variance (fANOVA) method proposed by F. Hutter et al. [30] and implemented it in Python [43]. Although fANOVA is not a direct approach for selecting specific hyperparameter values, it offers important scores for each hyperparameter. This information is valuable in narrowing down the range of hyperparameters by evaluating the marginal impact of different hyperparameter dimensions on the classification outcomes of the machine learning model.

Our objective was to reduce the hyperparameter space and identify the key hyperparameters that significantly influence the performance of the CNN, irrespective of the dataset used for training. Furthermore, fANOVA enables the identification of hyperparameters that have minimal or no impact on the model's performance. This knowledge obtained from the fANOVA analysis allows us to reduce the number and range of optimized hyperparameters, thereby minimizing the number of experiments, computational load, and time required.

To gather data for the fANOVA analysis, we trained CNN 20 times using all labeled images from each of the three datasets (Table 1). The classification performance of 60 CNNs was then evaluated based on accuracy. For each training session, the initial set of hyperparameters was randomly generated within the same predefined space for all datasets (see Table 2). The term *“number of fine-tuned CNN layers”* refers to the count of layers starting from the end of the network (excluding the final classification layer). For instance, if the number of fine-tuned layers is nine, it indicates that the last nine layers were retrained while the weights of the remaining layers were kept frozen.

### 3.2 Comparison of optimization techniques

To compare optimization techniques, we initially utilized the fANOVA results to determine the common ranges of hyperparameters for all four techniques (Table 2). Based on the fANOVA results (Fig. 3), we inferred that the best CNN performance is achieved with an input image size of 256 × 256 pixels and a training batch size of 16 images. Consequently, these two hyperparameters were fixed before proceeding with the comparison of optimization methods. As a result, image resolution and batch size were excluded from further optimizations.

To ensure reasonable analysis times and accommodate modest computational resources, we constrained the dropout factor range, the number of gradient descent optimization techniques, and the learning rate range for the Grid Search technique. These constraints allowed for a similar computational budget allocation across each optimization method. Additionally, we established specific parameters for ASHA (see Table 3) to prevent exceeding computational resource limits. These parameters were determined based on prior work (Hutter et al., 2014). Subsequently, the remaining hyperparameters (four out of six) common to all optimization techniques were optimized, and their corresponding search ranges are presented in Table 2B.

**Table 3:** Parameters and their values used by the ASHA method, where: r—is the min. number of training epochs, R—is the max. number of training epochs, *η*—is the elimination coefficient, s—is the early stopping criterion, and *N*_*m*_—is the number of fully trained models.

	Parameter				
	R	r	*η*	s	*N*_*m*_
Value	16	1	4	0	4

In each optimization run, the CNN was trained and evaluated using the same data split for training, validation, and testing (Table 1). To compare the optimization methods, we selected the hyperparameter configurations that yielded the highest CNN accuracy on the validation set.

### 3.3 Hyperparameter optimization with a subset of the training set

In this experiment, we aimed to investigate how hyperparameter optimization could be affected by three factors: (a) using a subset of the training set instead of the complete set, (b) the presence of a class imbalance in the training data, and (c) the utilization of class balancing methods to mitigate the imbalance. To conduct these experiments, we utilized both class-balanced and class-imbalanced subsets of the MIO-TCD dataset, each containing 19,250 images (Table 1), and employed the ASHA optimization method. CNN was trained separately using the balanced set, the original imbalanced dataset, and an augmented imbalanced dataset. Additionally, class weighting was implemented as an additional approach to mitigate the effect of class imbalance, applied to the last layer of the CNN during training. The performance of the CNN trained using the balanced set served as the reference baseline.

For the augmented imbalanced dataset, three perturbations were applied to each image, including rotation with a random angle between 0 and 10°, random zooming in the range of -10% to +10%, and vertical flipping. This resulted in three altered copies of each input image, in addition to the original images, effectively expanding the dataset comprising 4× as many images as the dataset before augmentation.

Class weighting was determined using the following formula:

(1) $$\begin{array}{c} w_{k}=ln\frac{n}{n_{k}}\; \end{array}$$

where: $w_{k}$ is the weight per class, $n_{k}$ is the number of images in class, and *n* is the number of all images.

Weights were calculated for all classes except the most common class (cars), for which the weight was set to 1. The weights for the other classes were set to values larger than 1.

These experiments were carried out using the ASHA optimization method because, in the previous experiments (Section 3.2), the selection of hyperparameters with ASHA consistently resulted in the highest accuracies across all investigated datasets. The five ASHA-specific parameters were set as outlined in Table 3 except for the number of trained models (parameter $N_{m}$) which was limited to 2.

**Figure 2:**
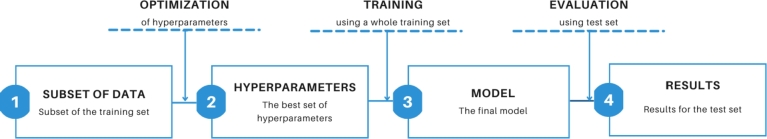
The main steps of the hyperparameter optimization using a subset of the training set.

The experiments were performed according to the scheme presented in Fig. 2. In the first step, optimal hyperparameters were determined using a subset of the training set. Subsequently, using the best set of hyperparameters, the final model was trained and evaluated.

## 4 Results

In our experiments, we utilized three publicly available datasets and evaluated CNN's performance by optimizing the training hyperparameters using four selected optimization methods. To thoroughly analyze the impact of hyperparameter selection, we performed a comprehensive research study that encompassed the following steps: (I) assessment of the hyperparameters' importance using fANOVA, and investigation of the impact of hyperparameter modifications on method performance, (II) comparing four optimization techniques, and (III) investigating the impact of training dataset size on hyperparameter optimization. The results obtained from our experiments are presented below.

### 4.1 Study I: assessment of the hyperparameters' importance using fANOVA

The assessment of the impact of hyperparameter selection on classification accuracy was conducted using the fANOVA library [30]. The important scores obtained from fANOVA are presented in Table 4, where higher scores indicate a greater impact of a hyperparameter on CNN's accuracy. The top two results (0.187 and 0.171) are for the learning rate and input image size, whereas the bottom two scores were achieved by batch size and GD optimization technique. Detailed values of the important scores for each dataset can be found in the supplement (Table A.8).

**Table 4:** The fANOVA-based parameter importance without stratification per dataset.

Hyperparameters	Parameter importance
Learning rate	0.187
Input image size	0.171
Dropout	0.138
Number of fine-tuned layers	0.037
Batch size	0.029
GD optimization technique	0.025

The fANOVA scores allow us to assess the impact of individual hyperparameters or groups of hyperparameters on classification accuracy. Based on these scores, we selected the hyperparameters with the top two important scores to further investigate their influence. Table 5 presents the top two hyperparameters for each examined dataset, while more detailed results can be found in Table A.8 in supplementary materials. The top two hyperparameters identified across the datasets were the input image size and the learning rate.

**Table 5:** Top hyperparameters based on the importance score, where * is the imbalanced development subset.

Dataset	The most important parameter pairs	
	Set I	Set II
Stanford Dogs	Optimization method & Input image size	Number of fine-tuned layers & Input image size
CIFAR-100	Optimization method & Number of fine-tuned layers	Number of fine-tuned layers & Input image size
MIO-TCD*	Optimization method & Dropout	and Dropout & Number of fine-tuned layers
All datasets	Learning rate & Input image size	Learning rate & Dropout

**Figure 3:**
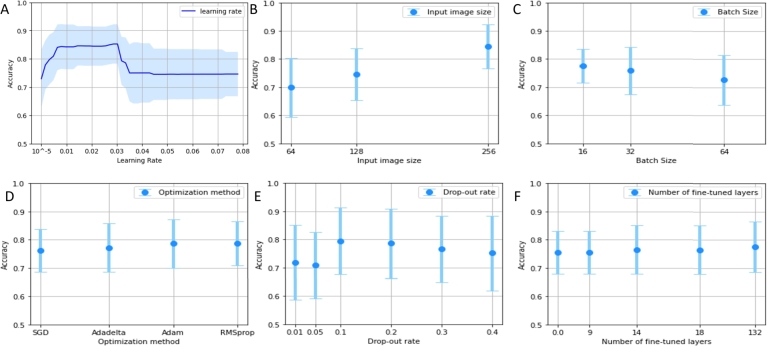
Impact of the value of select hyperparameters on the classification accuracy, where: A—learning rate; B—input image size; C—batch size; D—optimization method and F—number of fine-tuned layers.

Fig. 3 presents plots of the CNN's accuracy as a function of a single hyperparameter. From Fig. 3B, it can be inferred that increasing the image size leads to a monotonic increase in the CNN's accuracy. Since the highest accuracy (0.84) is achieved with the largest image size (256 × 256 pixels), it was decided to exclude image size from further optimizations. The plot in Fig. 3C indicates that batch size has minimal effect on the CNN's accuracy, with a maximum difference of 0.05 between means. This low impact is consistent with the low importance score of the batch size hyperparameter (Table 4). Consequently, any batch size within the predefined range could be used in subsequent experiments. However, to expedite image fetching from storage, a batch size of 16 was selected.

Remaining plots in Fig. 3 suggest that:

• Lower values of the learning rate correspond to the higher model performance (Fig. 3A), which aligns with existing literature [44], [45], [46]. Through our experiments, a learning rate value of 0.03 yielded the highest accuracy for the CNN model.
• It is not possible to estimate which optimization method had a greater impact on model performance based on the criterion used (Fig. 3D). The mean values for the investigated methods ranged from 0.76 to 0.79. However, further research could include an analysis of time as a factor, as differences might be expected in that regard.
• The dropout parameter exhibited the greatest impact on model performance when its value ranged from 0.1 to 0.3 (Fig. 3E). Similar ranges have been recommended in the literature [47], [48]. For instance, increasing the dropout value from 0.05 to 0.1 resulted in a 9% improvement in results. However, detailed results in Table A.8 of the supplementary materials demonstrate that the importance of this parameter varies based on dataset complexity.

The analysis of the impact of the number of fine-tuned layers on CNN model performance (Fig. 3.F) reveals that retraining the entire model produces similar results to retraining only the last 18 layers. This highlights the effectiveness of transfer learning and suggests that computational resources can be optimized by focusing on training a limited number of layers. Retraining more layers involves changing more model parameters, which increases computational requirements and training time. Determining the optimal number of layers to be retrained depends heavily on the dataset (refer to Table A.8 in the supplement for details).

### 4.2 Study II: comparison of four optimization techniques using three datasets

Table 6 presents a comparison of the four hyperparameter optimization techniques. Accuracy, calculated using the validation set, was used as the evaluation metric. To ensure a fair comparison, an equal research budget in terms of the total number of training epochs was allocated for all methods. The table displays the best results achieved by each method.

**Table 6:** Classification accuracy of the CNN with hyperparameters optimized by four methods. The highest accuracies within each dataset are bolded, where * indicates an imbalanced development subset, whereas each of the test sets was balanced.

Dataset	Method				Max difference
	Grid Search	Random Search	ASHA	Bayesian Optimization	
CIFAR-100	77.3%	78.5%	**83.0%**	81.6%	5.7%
MIO-TCD*	96.6%	96.5%	**97.2%**	96.8%	0.7%
Stanford Dogs	85.1%	85.1%	85.4%	**85.9%**	0.8%

The most significant difference in results can be observed for the CIFAR-100 dataset, where the application of the ASHA method led to a 6% improvement compared to the grid search method. For the other two datasets, each optimization method yielded similar results, with differences below 1%.

### 4.3 Study III: hyperparameters optimization with a subset of the training set

To explore whether hyperparameters can be effectively established using a subset of a training set, the same model was fine-tuned using different subsets: a balanced subset, an imbalanced subset, and an imbalanced subset with additional modifications such as class weighting and augmentation. For each case, the best hyperparameter settings were determined (Table 7), and training was then conducted using the entire training set. The evaluation was performed on the same test set. The results obtained are summarized in Table 7.

**Table 7:** Results achieved for sets of hyperparameters established using a subset of a training set whereas, the same, balanced test set was used in all cases. Marked by bolded font are the highest results.

Hyperparameter optimization dataset	Configuration				Result
	Learning rate	Dropout	Optimization method	Number of fine-tuned layers	Accuracy on the test set
Balanced	0.0249	0.1941	Adam	132	**85%**
imbalanced	0.0103	0.3861	RMSprop	14	76%
imbalanced & class weighting	0.0158	0.1856	Adam	132	**86%**
imbalanced & augmentation	0.0014	0.2837	RMSprop	132	83%
imbalanced & class weighting & augmentation	0.0056	0.3621	Adam	132	**85%**

whole training set	0.0162	0.2333	Adadelta	132	81%

If a successful hyperparameter search can be performed using a subset of the dataset, it offers several advantages. It allows for investigations with lower resource requirements, reducing the carbon footprint of the research by adopting responsible AI practices. It also decreases experimentation time and enables faster delivery of results or minimum viable product (MVP). The achieved results (Table 7) indicate two successful strategies: (I) using a balanced subset of the dataset, which resulted in a 4% increase in accuracy, and (II) utilizing an imbalanced data subset with additional modifications such as class weighting and augmentation, leading to a 4–5% improvement in results.

## 5 Discussion

Evaluation of results in Table A.8 indicates that the importance of individual parameters varies depending on the dataset. This highlights the necessity of evaluating parameter importance individually for each dataset.

The second experiment shows the comparison of the four tested hyperparameter optimization methods. Based on the achieved results, we could not identify a single method that consistently improved classification accuracy across all datasets. Instead, we have formulated the following conclusions:

• The ASHA and Bayesian methods outperformed the Grid and Random search techniques in terms of classification accuracy.
• The ASHA and Bayesian methods required fewer iterations to find hyperparameters which resulted in improved classification accuracy.
• For tasks with low complexity, the specific method used to determine the values of hyperparameters had a marginal impact on the results.

We can conclude that the application of ASHA or Bayesian Optimization techniques is applicable in the hyperparameter optimization tasks because they do not need to rely on prior knowledge to deliver a set of hyperparameters that improves the classification accuracy, even slightly. Although prior knowledge about dependencies between hyperparameters is not required to find the hyperparameters through the Grid and Random Search techniques, it may help narrow down the search space and, consequently, reduce the computational burden required to perform a more exhaustive search in the hyperparameter space.

In the third experiment, we investigated if hyperparameters can be optimized using a subset of available images and how operations such as class weighing and augmentation impact hyperparameter optimization. The obtained results are interesting because they demonstrate how datasets of various compositions used for hyperparameter optimization impact CNN's performance. Since the optimization of hyperparameters using all available images can be time-consuming, it is desirable to involve a reduced set of images to reduce the computational cost and time of the optimization process. The achieved results clearly indicate that balancing the training data is crucial in the optimization task. This balance can be achieved by either (I) extracting a balanced subset from the whole set of images or (II) extracting an imbalanced subset and then performing operations such as class weighing or class weighting and augmentation to reduce the imbalance. As a result, an application of a subset of the dataset that is balanced, either naturally or artificially through additional operations, allows for the establishment of the best hyperparameter set.

## 6 Conclusion

Indeed, hyperparameter settings play a crucial role in DL model training, and there are no universal baseline or default parameter values that guarantee optimal results for every research task. The default parameters might not be ideal for every image classification problem.

Manual selection of hyperparameters can be challenging and suboptimal, which is why parameter optimization methods like Grid Search and Random Search are valuable tools that can outperform manual selection. These methods allow users to explore the hyperparameter space more systematically and often yield better results compared to manual selection.

Prior knowledge and expertise can be leveraged to narrow down the hyperparameter space and reduce the optimization time. Experts can provide insights into the dependencies between hyperparameters, enabling a more focused search. However, it is important to note that hyperparameter optimization and importance assessment should be performed individually for each dataset, as the impact and significance of different hyperparameters can vary depending on the specific characteristics of the dataset.

The experiments conducted in this research demonstrate that hyperparameter optimization using ASHA or Bayesian Optimization techniques can effectively boost the classification accuracy of CNN models. Additionally, class balancing is crucial in hyperparameter optimization and using balanced datasets can significantly reduce the time required for hyperparameter optimization.

The research conducted on hyperparameter optimization for fine-tuned models creates opportunities for further investigations and studies. The studies can be conducted in the following areas: (I) investigating the impact of a bigger set of hyperparameters (in the paper, six hyperparameters were analyzed), (II) investigating and comparing other optimization techniques, such as genetic algorithms, (III) investigate the impact of optimization across different type of DL models (one, the state-of-the-art architecture was used in the study).

## Funding sources

The research was partially funded by the Centre for Priority Research Area Artificial Intelligence and Robotics of Warsaw University of Technology within the Excellence Initiative: Research University (IDUB) program. The funders had no role in the design of the study; in the collection, analyses, or interpretation of data; in the writing of the manuscript, or in the decision to publish the results.

## CRediT authorship contribution statement

**Mikolaj Wojciuk:** Writing – original draft, Visualization, Validation, Methodology, Investigation, Formal analysis, Data curation, Conceptualization. **Zaneta Swiderska-Chadaj:** Writing – review & editing, Writing – original draft, Supervision, Methodology, Funding acquisition, Conceptualization. **Krzysztof Siwek:** Funding acquisition, Conceptualization. **Arkadiusz Gertych:** Writing – review & editing, Writing – original draft, Supervision, Methodology.

## Declaration of Competing Interest

The authors declare that they have no known competing financial interests or personal relationships that could have appeared to influence the work reported in this paper.

## Data Availability

Used datasets are publicly available: (I) CIFAR-100 [38], (II) Stanford Dogs [39], and (III) MIO-TCD [40].

## Appendix A Supplementary material

MMC The supplementary material includes: (I) figure presented the Xception CNN architecture, and (II) table with ANOVA parameter importance stratified per dataset table. MMC
