# Supplementary material for: Improving classification accuracy of fine-tuned CNN models: Impact of hyperparameter optimization
Source: Heliyon. 2024 Feb 23;10(5):e26586. doi: 10.1016/j.heliyon.2024.e26586 (PMC10920154; doi:10.1016/j.heliyon.2024.e26586)
Supplement: MMC — The supplementary material includes: (I) figure presented the Xception CNN architecture, and (II) table with ANOVA parameter importance stratified per dataset table. [file mmc1.pdf]

Appendix A. Supplementary materials

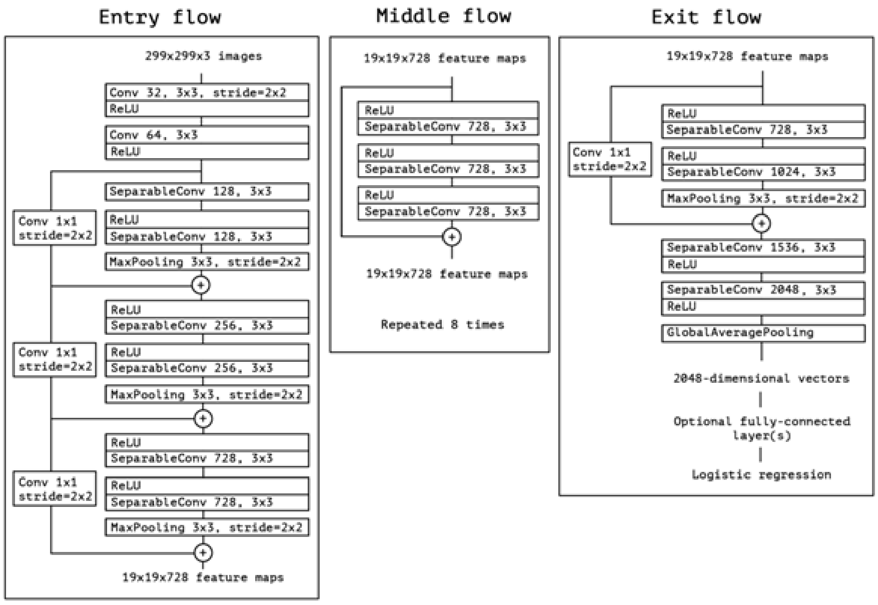

Figure A.4. The Xception CNN architecture is presented in [41]. In the study, the last layer was replaced by the softmax layer.

Table A.8. fANOVA parameter importance stratified per dataset

	**Hyperparameter**					
Dataset	Optimization method	Learning rate	Dropout	Number of fine-tuned layers	Input image size	Batch size
Stanford Dogs	0.015	0.089	0.004	0.148	0.681	0.038
CIFAR-100	0.192	0.063	0.138	0.117	0.229	0.024
Subsets of MIO-TCD	0.089	0.378	0.268	0.128	0.004	0.060
